# Multiple endocrine neoplasia type 1: extensive analysis of a large database of Florentine patients

**DOI:** 10.1186/s13023-018-0938-8

**Published:** 2018-11-14

**Authors:** Francesca Marini, Francesca Giusti, Maria Luisa Brandi

**Affiliations:** Department of Surgery and Translational Medicine, University of Florence, Largo Palagi 1, 50139 Florence, Italy

**Keywords:** Multiple endocrine neoplasia type 1 (MEN1), *MEN1* gene, Genetic test, Primary hyperparathyroidism (PHPT), Gastro-entero-pancreatic neuroendocrine tumours (GEP-NETs), Pituitary adenomas, Patients’ database

## Abstract

**Background:**

Multiple endocrine neoplasia (MEN1) is a rare inherited multi-tumour syndrome, affecting specific neuroendocrine organs and non-endocrine tissues with a variable spectrum of over 20 possible different combinations, caused by inactivating heterozygote mutations of the* MEN1* gene.

Disease onset, penetrance, clinical presentation, course and prognosis are all extremely variable, even among individuals bearing the same causative mutation, which doesn’t allow prediction of the individual clinical phenotype (based on the specific result of the genetic test), thus compelling all patients and mutation carriers to undergo a common routine general screening program.

**Results:**

We performed an extensive epidemiological, clinical and genetic analysis of the Florentine MEN1 patient database, which includes 145 MEN1 patients and 20 asymptomatic *MEN1* carriers, constantly followed up at the Regional Referral Centre for Inherited Endocrine Tumours of the Tuscany Region, during the last three decades. We reported, here, the results of clinical, epidemiological and genetic descriptive statistics, as well as correlation analyses between tumours and mutation types and localisation. No direct genotype-phenotype correlation was described, but the importance of the genetic testing was confirmed for an early diagnosis and the identification of asymptomatic carriers.

**Conclusions:**

As with all rare diseases, the possibility to collect and analyse data on a relatively large number of patients is important for increasing our knowledge of the epidemiologic aspects of the disease, and its natural course and prognosis of single manifestations of the syndrome, in order to set up the best diagnostic and therapeutic plans for patients. In this light, the creation and constant updating of large patient databases is fundamental. Results from database study can provide useful epidemiological, clinical and genetic information about MEN1 syndrome, which could help clinicians in the diagnostic and therapeutic management of single MEN1 patients.

## Background

Multiple endocrine neoplasia (MEN1) is a rare (prevalence 3–20/100,000) congenital endocrine syndrome, consisting of the development of multiple neuroendocrine tumours (NETs) in a single patient, principally at parathyroid glands, anterior pituitary and gastro-entero-pancreatic (GEP) and thoracic tracts. Other endocrine and non-endocrine tissues can be affected with less frequency, accounting for over 20 different combinations of tumours and lesions.

Principally affected organs are the parathyroids; parathyroid multiple adenomas affect up to 100% of patients by the age of 50, representing the first clinical manifestation in about 90% of cases with a mean age of onset of 20–25 years and rare described cases also by the age of 8 [1]. Parathyroid disease manifests principally as primary hyperparathyroidism (PHPT), which can be normocalcemic in some cases, and often remains asymptomatic in many patients. Symptomatic PHPT is due to the prolonged hypercalcemia and it usually results in loss of bone mass (osteopenia or osteoporosis) and/or nephrocalcinosis. Adenoma ablation by surgery is the treatment of choice, mostly for hypercalcemic and symptomatic cases, even if both the right time and the kind of intervention are still controversial. Pharmacological treatment with calcimimetic drugs demonstrated to be able to control hypercalcemia in patients who do not meet the criteria for parathyroid surgery or do not want to undergo the intervention [2].

The second most common manifestation in MEN1 are neuroendocrine tumours of the GEP tract (GEP-NETs), affecting about 30–70% of patients [1], usually manifesting as multiple adenomas and often recurrent after surgical resection. They present both as micro- or macro-adenomas (diameter over 0.5 cm), and can be non-functioning tumours (NFTs; 20% of GEP-NETs) or active tumours secreting excessive quantities of hormones. NFTs are located mainly in the pancreas and usually they remain asymptomatic till their excessive growth causes compression of adjacent tissues and/or their (frequent) malignant progression and metastases manifest. A continuous imaging surveillance is required and surgery ablation is suggested for NFTs over 0.5 cm in diameter. Recently, also non-functioning NETs (NF-NETs) of the stomach (previously referred as gastric carcinoid of type II enterochromaffin-like cells) have been included in the GEP-NETs group; they have an estimated penetrance of about 10% of MEN1 patients. Functioning GEP-NETs produce excessive hormones, such as gastrin, insulin, somatostatin, glucagon or vasoactive intestinal polypeptide (VIP), causing in many cases an associated endocrine syndrome, and they are, respectively, named gastrinomas, insulinomas, somatostatinomas, glucagonomas and VIPomas. Gastrinomas are the most common (over 50% of cases) MEN1 secreting GEP-NETs; about 90% are located in the duodenum and 10% in the pancreas. Pancreatic gastrinomas are more aggressive. Over secretion of gastrin is responsible for Zollister Ellison Syndrome (ZES) in many cases. Usually, MEN1 gastrinomas are small (less than 0.5 mm) and multiple, with a frequent rate of malignant progression and metastases development, being, together with severe ulcers, one of the most common causes of MEN1-related premature deaths. Insulinomas are the second most common GEP-NETs in MEN1, manifesting in 10–30% of patients, often as multiple tumours. Surgery is usually the therapeutic approach to GEP-NETs, but it is not always effective due to multiple nature of these tumours that are often scattered through the entire neuroendocrine tissue. In case of non-resectable tumours or advanced metastatic cancer, some pharmacological treatments demonstrated to be effective in increasing median progression-free survival [i.e somatostatin analogues (SSAs), cytotoxic chemotherapy (streptozocin and 5-fluorouracil, doxorubicin, temozolomide with capecitabine), inhibitors of thyrosin kinase receptors (sunitinib), and inhibitors of the mammalian target of rapamycin (everolimus)].

Adenomas of anterior pituitary gland affect about 30–40% of MEN1 patients and represent the third most common tumours in MEN1. They can be hormone-secreting or NFTs. Often they develop as single tumours, are non-invasive, and very rarely manifest a malignant progression. Signs and symptoms are principally due to macro-adenomas compressing cerebral structures, or specifically derived by the over-production of one or more pituitary hormones (i.e prolactin, PRL; somatotropin, GH; and corticotropin, ACTH). Functioning tumours are prolactinomas (PRLomas, 60% of pituitary adenomas), somatotropinomas (25%) or corticotropinomas (5%). Trans-sphenoidal or endoscopic resection or radioablation are the treatments of choice for macro-adenomas and NFTs. PRL-secreting micro-adenomas are pharmacologically treated by dopamine agonists, while somatotropinomas are treated with SSAs.

Other MEN1-associated NETs are thoracic carcinoids, of the thymus and the bronchopulmonary tract, in 3% of cases, and tumours/lesions of the adrenal glands in about 20–40% of patients.

Non-endocrine multiple skin lesions are also frequent (i.e collagenomas, angiofybromas, fibromas, angiomas, and lipomas), often manifesting even before of MEN1 neuroendocrine tumours and being, thus, useful in favoring an early diagnosis. Lipomas can manifest also at visceral level.

Germinal inactivating heterozygote mutations of the *MEN1* tumour suppressor gene have been identified as responsible for the development of the syndrome, mostly through the loss of the second wild type copy of the gene at somatic level of specifically predisposed neuroendocrine tissues. To date, over 1500 different germinal and somatic mutations, spanning the entire coding region (exons 2–10) and splicing sites of *MEN1*, have been described, none of them being associated with a specific clinical phenotype and/or disease penetrance [3, 4]. The lack of a direct genotype-phenotype correlation does not allow to foresee the exact clinical course and tumour localization of the disease, to program personalised diagnostic screening or therapeutic plans.

Here we performed an extensive epidemiological, clinical and genetic analysis of the wide Florentine MEN1 patients’ database, which includes MEN1 patients and asymptomatic *MEN1* carriers constantly followed up at the Regional Referral Centre for Inherited Endocrine Tumours of the Tuscany Region, during the last three decades.

## Materials and methods

### Patients

Patients included in this study have been clinically following up at the Ambulatory of the Regional Referral Centre for Inherited Endocrine Tumours of the Tuscany Region, at the “Azienda Ospedaliero-Universitaria Careggi, Firenze” from 1991 to date. The clinical and genetic study was approved by the Internal Review Board of the “Azienda Ospedaliero-Universitaria Careggi, Firenze”. All patients enrolled in the study, or their legal tutors in case of patients less than 18 years, were requested to sign an informed consent form prior their data were included in the analysis. Collected data were appropriately made anonymous and each patient was identified by a unique alphanumeric identification code; data were all analysed as aggregates.

We included in this study a total of 165 MEN1 patients [59 males (35.8%) and 106 females (64.2%)], of which 27 are referred as simple cases and 138 as familiar cases (from 51 different pedigrees). Familial cases were defined when at least two MEN1 clinical cases are identified within a family or when at least two family members bear a *MEN1* mutation.

We collected, in a specific computed database, data about gender, date of birth, familial and personal clinical history (i.e. age at clinical and genetic diagnosis, type and age of onset of the first clinical manifestation, all endocrine and non-endocrine MEN1-associated manifestations and related signs and symptoms, past and present MEN1 therapies, all surgical MEN1 interventions) and the result of the *MEN1* genetic test.

According to International guidelines, the MEN1 diagnosis was established based on one of these three criteria: 1) presence of tumours in at least two of the three main organs/tissues affected in MEN1, 2) presence of tumours in one of the three main organs/tissues affected in MEN1 and a first-degree relative affected by MEN1, 3) the identification of a germinal inactivating mutation of the *MEN1* gene.

Age at diagnosis was considered the age at which the syndrome was definitely recognised; for each patient we considered an age at clinical diagnosis (recognition of MEN1 by clinical signs and/or symptoms) and an age at genetic diagnosis (age of genetic identification of a *MEN1* mutation).

PHPT was classified as symptomatic only when patients manifested secondary signs and/or symptoms due to prolonged hypercalcemia, such as nephrolithiasis and/or secondary osteopenia and osteoporosis; patients with elevated parathyroid hormone (PTH), with or without hypercalcemia, but without any associated secondary clinical signs were considered asymptomatic PHPT.

GEP-NETs were classified as non-functioning if they did not secrete hormones or secreted only neuroendocrine polypeptides which do not cause a specific clinical syndrome [i.e chromogranin A and pancreatic polypeptide (PP)].

Patients not showing any clinical sign and/or symptom associated with MEN1 at the time of this study were considered as asymptomatic and they were excluded from the analysis of genotype-phenotype association.

### *MEN1* genetic analysis

*MEN1* genetic screening for mutations was performed in all the 165 patients of our database by PCR-based Sanger’s sequencing of genomic DNA from blood. We analysed the coding region (exons 2–10) and the splicing-sites of the gene; obtained sequences were compared to the human wild type reference sequence of the *MEN1* gene (OMIM 613733); mutations were classified using the standard nomenclature for human DNA sequence variants. When a mutation was identified in a MEN1 index case, the genetic analysis for this specific mutation was extended also to first degree relatives. Among the 16 MEN1 patients resulted negative by Sanger’s sequencing, 6 patients (from 3 different families) were further investigated. One family was analysed by microsatellite-based haplotype linkage analysis at 11q13 locus. The other two families were screened (by two external laboratories) by multiplex ligation-dependent probe amplification (MPLA) to identify large intragenic deletions/insertions.

### Statistical analyses

Clinical manifestations, age at diagnosis, therapies (surgical and pharmacological), mutation distribution and classification were all analysed by descriptive statistics; data are presented as nominal categories, percentages or mean + standard deviation (SD).

Correlations between clinical data and mutation type and/or mutated gene region were analysed by chi-squared test, assuming a positive significance with *p* < 0.05 (Yates’ correction was applied for subgroups containing less than 5 cases). Only the four main mutation types (frameshift, nonsense, missense and splicing site) and only mutations at exons 2, 3, 9, 10 and intron 4 were included in the comparative analyses, since double mutation, large intragenic deletions, predisposing haplotype, absence of an identified mutation, and mutations located in all the other exons/introns were all carried by a very little number of patients (< 10) to be able to carry on a sufficiently strong association statistical test.

## Results

### Patients

Of the 165 MEN1 patients included in the database, 145 were clinically affected at the time of this study, while 20 showed no signs of the disease (asymptomatic mutation carriers). PHPT, GEP-NETs and pituitary tumours resulted to be the three most common clinical manifestations in affected subjects, respectively in 139 (95.86%), 86 (59.31%) and 75 (51.72%) cases. Detailed prevalence of MEN1 tumours/lesions in our series is reported in Table 1, in comparison to data reported in the latest MEN1 guidelines [1].

**Table 1:** Prevalence of MEN1 tumours/lesions in our series with respect to published data*

	Prevalence in our patients n. (%)	Type and combination of tumours/lesions in our patients n. (%)	Prevalence in published literature (%)
PHPT	139 (95.86%)	139 PHPT:	90%
		- 85 asymptomatic cases (61.15%)	
		- 36 with nephrocalcinosis (25.90%)	
		- 7 with osteoporosis (5.03%)	
		- 7 with nephrocalcinosis and osteoporosis (5.03%)	
		- 2 with osteopenia (1.44%)	
		- 2 with nephrocalcinosis and osteopenia (1.44%)	
GEP-NETs	86 (59.31%)		30–70%
		100 total tumours:	
		- 41 gastrinomas (28.27% of MEN1 affected patients; 41% of GEP-NETs)	40%
		- 16 insulinomas (11.03% of MEN1 affected patients; 16% of GEP-NETs)	10%
		- 39 pNFTs (including 1 PPoma) (26.90% of MEN1 affected patients; 39% of GEP-NETs)	20–55%
		- 2 VIPomas (1.38% of MEN1 affected patients; 2% of GEP-NETs)	< 1%
		- 1 glucagonoma (0.69% of MEN1 affected patients; 1% of GEP-NETs)	< 1%
		- 1 gastric NF-NET (0.69% of MEN1 affected patients; 1% of GEP-NETs)	10%
		Combinations:	
		- 28 gastrinoma alone (32.56% of patients affected by GEP-NETs)	
		- 13 insulinoma alone (15.12% of patients affected by GEP-NETs)	
		- 29 pNFTs alone (including 1 PPoma) (33.72% of patients affected by GEP-NETs)	
		- 2 VIPoma alone (2.33% of patients affected by GEP-NETs)	
		- 9 gastrinoma-pNFTs (10.47% of patients affected by GEP-NETs)	
		- 3 gastrinoma-insulinoma (3.49% of patients affected by GEP-NETs)	
		- 1 gastrinoma-glucagonoma (1.16% of patients affected by GEP-NETs)	
		- 1 pNFT-gastric NF-NET (1.16% of patients affected by GEP-NETs)	
Pituitary tumours	75 (51.72%)		30–40%
		76 total tumours:	
		- 60 PRLomas (41.38% of MEN1 affected patients; 78.95% of pituitary tumours)	20%
		- 12 NFA (8.28% of MEN1 affected patients; 15.79% of pituitary tumours)	< 5
		- 3 ACTH-secreting tumours (corticotropinomas) (2.07% of MEN1 affected patients; 3.95% of pituitary tumours)	< 5%
		- 1 GH-secreting tumour (somatotropinoma) (0.69% of MEN1 affected patients; 1.32% of pituitary tumours)	10%
		Combinations:	
		- Only one combination of PRLoma-somatotropinoma (1.33% of patients affected by pituitary adenoma)	
Carcinoids	17 (11.72%)	17 bronchopulmonary (11.72% of MEN1 affected patients)	Bronchopulmonary NETs 2% Thymic NETs 2%
Skin lesions	44 (30.34%)	53 total skin lesions:	30%
		- 37 lipomas: 17 single lipomas and 20 multiple lipomatosis (25.52% of MEN1 affected patients; 69.81% of skin lesions)	85%
		- 9 angiofibromas (6.21% of MEN1 affected patients; 16.98% of skin lesions)	n.a
		- 4 angiomas (2.76% of MEN1 affected patients; 7.55% of skin lesions)	n.a
		- 3 fibromas (2.07% of MEN1 affected patients; 5.66% of skin lesions)	
Adrenocortical tumours/lesions	27 (18.62%)		40%
		27 total tumours/lesions of adrenal glands:	
		- 9 hyperplasia (5 monolateral, 4 bilateral) (6.21% of MEN1 affected patients; 33.33% of adrenal gland tumours/lesions)	
		- 18 adenomas (15 monolateral, 3 bilateral) (12.41% of MEN1 affected patients; 66.67% of adrenal gland tumours/lesions)	
Other lesions	19 (13.10%)		n.a.
		20 total other associated tumours:	
		- 3 meningiomas (2.07% of MEN1 affected patients)	8%
		- 4 breast cancers (4.21% of MEN1 affected women)	n.a
		- 12 uterine lesions (12.63% of MEN1 affected women)	n.a
		- 1 perineal aggressive angiomixoma (0.69% of MEN1 affected patients)	n.a.

Footnotes: *N* number, *PHPT* Primary hyperparathyroidism, *GEP-NETs* gastro-entero-pancreatic neuroendocrine tumours, *pNFTs* Pancreatic non-functioning tumours, *NF-NET* Non-functioning neuroendocrine tumour, *VIP* Vasoactive intestinal peptide, *PP* Pancreatic polypeptide, *PRLoma* Prolactinoma, *NFA* Non-functioning adenoma, *GH* Growth hormone (somatotropin), *ACTH* adreno-cortico tropic hormone (corticotropin), *N.A.* non-available

*Thakker et al. Clinical practice guidelines for multiple endocrine neoplasia type 1 (MEN1). J Clin Endcocrinol Metab 97(9): 2990–3011, 2012

Forty-six of the affected patients (31.72%) presented one of any clinical combination of the triad PHPT, GEP-NETs and pituitary adenomas. Most common phenotypical combinations were, in order of frequency: PHPT/GEP-NETs/pituitary tumours in 46 cases (31.72%), PHPT/GEP-NETs in 36 cases (24.83%), PHPT/pituitary tumours in 24 cases (16.55%) and GEP-NETs/pituitary tumours in 3 cases (2.07%). Distributions of clinical manifestations in our series of patients are depicted in detail in Table 2.

**Table 2:** Distribution and intra-tissue combinations of MEN1 main tumours in our series of MEN1 patients

Only PHPT n. (%)	Only GEP-NET n. (%)	Only pituitary adenoma n. (%)	PHPT/GEP-NETs/Pituitary n. (%)	PHPT/GEP-NETs n. (%)	PHPT/pituitary adenomas n. (%)	GEP-NETs/pituitary adenomas n. (%)	PHPT not associated with classical MEN1 main tumours n. (%)	Asymptomatic n.	Total patients n.
23 (15.86%) - 19 asymptomatic PHPT (13.10%) - 2 PHPT with osteoporosis (1.38%) - 1 PHPT with nephrocalcinosis - 1 PHPT with nephrocalcinosis and osteoporosis	1 (0.69%) - 1 pNFTs	2 (1.38%) - 2 PRLomas	46 (31.72%) - 10 PHPT/gastrinoma/PRLoma (6.90%) - 8 PHPT/insulinoma/PRLoma (5.52%) - 1 PHPT/insulinoma/pituitary NFA 0.69%) - 14 PHPT/pNFTs/PRLoma (9.66%) - 4 pHPT/pNFTs/pituitary NFA (2.76%) - 2 PHPH/VIPoma/PRLoma (1.38%) - 1 PHPT/insulinoma/gastrinoma/corticotropinoma (0.69%) - 3 PHPT/gastrinoma/pNFTs/PRLoma (2.07%) - 2 PHPT/gastrinoma/pNFTs/pituitary NFA (1.38%)	36 (24.83%) - 13 PHPT/gastrinoma (8.97%) - 12 PHPT/pNFTs (8.28%) - 4 PHPT/gastrinoma/pNFTs (2.76%) - 2 PHPT/gastrinoma/insulinoma (1.38%) - 1 PHPT/gastrinoma/glucagonoma (0.69%)	24 (16.55%) - 17 PHPT/PRLoma (11.72%) - 5 PHPT/pituitary NFA (3.45%) - 1 PHPT/corticotropinoma (0.69%) - 1 PHPT/PRLoma/somatotropinoma (0.69%)	3 (2.07%) - 3 gastrinoma/PRLoma (2.07%)	10 (6.90%) - 3 PHPT with skin lesions (2.07%) - 4 PHPT with uterine myoma (2.76%) - 2 PHPT with adenoma of adrenal glands (1.38%)	20	165

Footnotes: *N*. Number, *PHPT* Primary hyperparathyroidism, *GEP-NETs* Gastro-entero-pancreatic neuroendocrine tumours, *pNFTs* Pancreatic non-functioning tumours, *PRLoma* Prolactinoma, *NFA* Non-functioning adenoma, *VIP* Vasoactive intestinal peptide. Percentages were calculated with respect to MEN1 clinically affected patients (145)

Mean age of the first clinical manifestation was 31.8 + 13.5 years (range 9–71 years).

Mean age at diagnosis for index cases (both single and familial) was 40.6 + 15.6 years (range 4–73 years). Age at genetic diagnosis for relatives of the index cases was 31.2 + 16.9 years (range 1–71 years), with a positive mean gap of about 10 years in anticipating diagnosis with respect to probands. Twenty individuals identified as mutation carriers resulted free of any MEN1 clinical sign or symptom at the time of the study and were considered as asymptomatic; they are still under constant MEN1 diagnostic surveillance, according to International guidelines [1].

Nine patients died (6.21% of affected patients) because of MEN1-related causes and malignant progression of MEN1 tumours. Mean age of death was 63.9 + 14.6 (range 37–88 years). Three died for liver metastases from gastrinoma, one for severe peptic ulcer, one for severe gastric bleeding, one for atrial fibrillation and hypokalemia, one for post-surgical hyponatremia for recurrent PRLoma, one for post-surgical complications of a recurrent lung carcinoid and one for a lung carcinoid and untreated ZES. Mean age of first clinical manifestation was 39.6 + 16.8 (range 17–63 years), mean age at MEN1 diagnosis was 56.6 + 9.8 years (range 35–71 years) with a gap between the first manifestation occurrence and the correct diagnosis of over 15 years.

### PHPT

One hundred thirty-nine patients were affected by PHPT (47 men and 92 women). Mean age at diagnosis of PHPT was 34.3 + 13.1 years (range 7–73 years), while mean age of MEN1 diagnosis, for these patients, was 35.1 + 15.3 years (range 7–73 years).

In 23 cases, PHPT was the only clinical manifestation (15.86% of MEN1 symptomatic affected patients; 16.55% of patients with PHPT), while in 116 cases PHPT was associated with other MEN1 tumours/lesions, as reported in Table 2.

PHPT was the first clinical manifestation in 92 cases (63.45% of all MEN1 affected patients; 66.19% of PHPT cases); 25 of them were diagnosed with PHPT after symptoms of nephrocalcinosis and/or renal colic, while 67 were biochemically diagnosed in presence of elevated serum PTH and, in the great majority of cases (65), also hypercalcemia. Mean age of PHPT discovery, in these 92 patients, was 34.4 + 13.5 (range 12–71 years). Fifty-two were index cases [mean age of PHPT discovery 33.0+ 12.5 (range 14–66 years)] and 40 were relatives of a MEN1 proband [mean age of PHPT discovery 36.2+ 14.5 (range 12–71 years)].

Ninety-nine PHPT affected patients underwent parathyroid surgery (71.22% of PHPT affected patients), while 40 did not undergo any parathyroid surgery (13 of them were treated with cinacalcet). Total parathyroidectomy (TPT) was the most performed surgical approach in our patients (47 cases; 33.81% in all PHPT affected patients and 47.47% of parathyroid surgical interventions); parathyroid tissue autograft in the non-dominant forearm was performed in 43/47 cases (91.49%). A percentage of both subtotal parathyroidectomy (SPT) and partial parathyroidectomy (PPT) required a second intervention for adenoma recurrences [2 cases for STP (11.76%) and 14 cases for PPT (40%)]: 10 TPTs (only one without tissue autograft), 2 SPTs and 4 PPTs. Five cases of permanent post-surgical hypoparathyroidism were reported (5.1% of all operated patients; 10.6% of TPT), all of them after a TPT.

Principal characteristics and treatments of PHPT in our series are reported in Table 3.

**Table 3:** Main characteristics and treatments of PHPT in our series of MEN1 patients

PHPT	Patients n. (%)	Clinical presentation		Surgery				Pharmacological treatment (Calcimimetic) n. (%)
		Symptomatic n. (%)	Asymptomatic n. (%)	No surgery n. (%)	TPT n. (%)	SPT n. (%)	PPT n. (%)	
Total patients with PHPT	139 (95.87%)	54 (37.24%)	85 (58.62%)	40 (28.78%)	47 (33.81%) - 43 with tissue autograft in the non-dominant forearm - 4 without tissue autograft	17 (12.23%)	35 (25.18%)	26 (18.70%)
PHPT as first clinical manifestation	92 (63.45%)	43 (29.66%) - 29 nephrocalcinosis (20%) - 7 nephrocalcinosis and osteoporosis (4.83%) - 5 osteoporosis (3.45%) - 2 nephrocalcinosis and osteopenia (1.38%)	49 (33.79%)	23 (16.55%)	29 (20.86%) - 28 with tissue autograft in the non-dominant forearm - 1 without tissue autograft	12 (8.63%)	28 (20.14%)	16 (11.51%)
Index cases (probands)	85 (58.62%)	37 (25.52%) - 26 nephrocalcinosis (17.93%) - 5 nephrocalcinosis and osteoporosis (3.45%) - 3 osteoporosis (2.07%) - 2 osteopenia (1.38%) - 1 nephrocalcinosis and osteopenia (0.69%)	48 (33.10%)	19 (13.67%)	33 (23.74%) - 30 with tissue autograft in the non-dominant forearm - 3 without tissue autograft	10 (7.19%)	23 (16.55%)	17 (12.23%)
Relatives	54 (37.24%)	16 (11.03%) - 9 nephrocalcinosis (6.21%) - 4 osteoporosis (2.76%) - 2 nephrocalcinosis and osteoporosis (1.38%) - 1 nephrocalcinosis and osteopenia (0.69%)	38 (26.21%)	21 (15.11%)	14 (10.07%) - 13 with tissue autograft in the non-dominant forearm - 1 without tissue autograft	7 (5.04%)	12 (8.63%)	9 (6.48%)

Footnotes: *PHPT* Primary hyperparathyroidism, *N* number, *TPT* Total parathyroidectomy (all four glands removed), SPT Subtotal parathyroidectomy (three glands and part of the forth gland removed), *PPT* Partial parathyroidectomy (three or less glands removed)

Percentages of clinical presentation were calculated with respect to MEN1 clinically affected patients (145). Percentages of surgery and pharmacological treatment were calculated with respect to PHPT affected patients (139)

Surgical interventions in Table 3 included only the first parathyroid surgery (14 PPTs and 2 SPTs required a second parathyroid intervention for recurrences: 10 TPTs, 2 SPTs and 4 PPTs)

### GEP-NETs

Eighty-six patients were affected by GEP-NETs (26 men and 60 women). Mean age at diagnosis of GEP-NETs was 40.1 + 13.1 (range 14–73 years), while mean age of MEN1 diagnosis, for these patients, was 33.6 + 12.8 (range 14–63 years).

Only one patient presented a GEP-NET (pancreatic NET; pNET) as the only clinical manifestation (0.69% of MEN1 symptomatic affected patients; 1.16% of patients with GEP-NETs); in all the other 85 cases GEP-NETs were associated with other MEN1 tumours/lesions, as reported in Table 2.

A GEP-NET was the first clinical manifestation in 20 individuals (13.79% of all MEN1 affected patients; 23.26% of GEP-NET cases); 9 of them were gastrinomas (of which 4 were diagnosed after manifesting ZES, 2 after presenting duodenal ulcer and 3 after gastric symptoms), 8 were insulinomas (of which 4 were diagnosed after manifesting constant hypoglycaemia and/or recurrent hypoglycaemic crisis), one was VIPoma (initially diagnosed by elevated serum VIP level), one was PPoma (initially diagnosed by elevated serum PP and chromogranin A levels) and one was a gastric NF-NET. Mean age of first GEP-NET biochemical or imaging discovery, in these 20 patients, was 30.9 + 12.2 (range 14–59 years). Fourteen were index cases [mean age of GEP-NET discovery 33.5+ 12.7 (range 14–59 years)] and 6 relatives of a MEN1 proband [mean age of GEP-NET discovery 23.6+ 6.2 (range 17–35 years)].

Forty-five patients with GEP-NETs underwent pancreas/duodenal surgery (52.33%% of GEP-NET affected patients). Partial pancreas resection or selective tumour enucleation were performed in 28 of all the GEP-NET-operated patients (32.56% of GEP-NET affected patients and 62.22% of GEP-NET interventions), while 17 patients underwent Whipple’s procedure (19.77% of GEP-NET affected patients and 37.78% of GEP-NET interventions). One patient was treated with SSAs-conjugated radionuclide therapy with (177)Lu-DOTATATE for non-resectable pNET, while another patient was firstly treated with SSAs, followed by four cycle of SSAs-conjugated radionuclide therapy with (177)Lu-DOTATATE before undergoing pancreaticoduodenectomy with partial liver resection.

Principal characteristics and treatments of GEP-NETs in our series are reported in Table 4.

**Table 4:** Main characteristics and treatments of GEP-NETs in our series of MEN1 patients

GEP-NETs	Patients n. (%)	Clinical presentation			Surgery			Pharmacological treatment	
		FTs n. (%)	NFTs (including PPoma) n. (%)	Combination of FT and NFT n. (%)	No surgery n. (%)	WP n. (%)	Enucleation or PPR n. (%)	(SSAs) n. (%)	Radionuclide therapy with (177)Lu-DOTATATE n. (%)
Total patients with GEP-NETs	86 (59.31%)	47 (32.41%) - 28 gastrinomas alone (19.31%) - 13 insulinomas alone (8.97%) - 2 VIPomas alone (1.38%) - 3 gastrinoma-insulinoma (2.07%) - 1 gastrinoma-glucagonoma (0.69%)	30 (20.69%) - 28 pNFTs (19.31%) - 1 PPoma (0.69%) - 1 pNFT-gastric NF NET (0.69%)	9 (6.21%) - 9 gastrinoma-PNFTs (6.21%)	41 (47.67%)	17 (19.77%)	28 (32.56%)	18 (20.93%)	2 (2.36%)
A GEP-NET as first clinical manifestation	20 (13.79%)	18 (12.41%) - 9 gastrinomas (6.21%) - 8 insulinomas (5.52%) - 1 VIPoma (0.69%)	2 (1.38%) - 1 PPoma (0.69%) - 1 gastric NF NET (0.69%)	0	5 (5.81%)	6 (6.98%)	9 (10.47%)	4 (4.65%)	1 (1.16%)
Index cases (probands)	65 (44.83%)	39 (26.90%) - 22 gastrinomas alone (15.17%) - 11 insulinomas alone (7.59%) - 2 VIPomas alone (1.38%) - 3 gastrinoma-insulinoma (2.07%) - 1 gastrinoma-glucagonoma (0.69%)	17 (11.72%) - 16 pNFTs (11.03%) - 1 pNFT-gastric NF NET (0.69%)	9 (6.21%) - 9 gastrinoma-PNFTs (6.21%)	25 (29.07%)	17 (19.77%)	23 (26.74%)	12 (13.95%)	2 (2.36%)
Relatives	21 (14.48%)	8 (5.52%) - 6 gastrinomas alone (4.14%) - 2 insulinomas alone (1.38%)	13 (8.97%) - 12 pNFTs (8.28%) - 1 PPoma (0.69%)	0	16 (18.60%)	0	5 (5.81%)	6 (6.98%)	0

Footnotes: *N* number, *FTs* Functioning tumours, *NFTs* Non-functioning tumours, *pNFTs* pancreatic non-functioning tumours, *NET* Neuroendocrine tumours, *WP* Whipple’s procedure (pancreaticoduodenectomy; removal of the head of the pancreas, the duodenum, the proximal jejunum, gallbladder, and part of the stomach), *PPR* Partial pancreas resection (partial pancreatoctomy), *SSAs* somatostatin analogues (octreotide or lanreotide)

Percentages of clinical presentation were calculated with respect to MEN1 clinically affected patients (145). Percentages of surgery and pharmacological treatments were calculated with respect to GEP-NET affected patients (86)

### Pituitary tumours

Seventy-five patients manifested at least one pituitary tumours (21 men and 54 women).

Mean age at diagnosis of pituitary tumours was 33.0 + 14.3 (range 7–69 years), while mean age of MEN1 diagnosis, for these patients, was 31.5 + 14.0 (range 7–57 years).

Two individuals (both index cases and non-familial cases), aged respectively 48 and 55 years at the time of this study, presented only a PRLoma as MEN1 manifestation (diagnosed at the age of 36 and 50 years, respectively). In all the other 73 cases, pituitary tumours were associated with other MEN1 tumours/lesions, as reported in Table 2.

PRLoma was the most common pituitary tumour (80%), affecting 60 individuals (one in association with somatotropinoma). A detailed distribution of pituitary tumours in our patients is depicted in Table 1.

A pituitary tumour was the first clinical manifestation in 25 patients: 24 were PRLomas (all of them were diagnosed by biochemical recognition of high serum level of PRL; 4 presented signs of amenorrhea, 1 of oligomenorrhea, 2 of galactorrhea, 1 of both oligomenorrhea and galactorrhea and 1 woman presented hypogonadism and androgenic phenotypical manifestations) and one corticotropinoma. Mean age of pituitary tumour discovery, in these 25 patients, was 26.7 + 12.3 (range 12–55 years). Eighteen were index cases [mean age of pituitary tumour discovery 30.6+ 12.6 (range 12–55 years)] and 7 relatives of a MEN1 proband [mean age of pituitary tumour discovery 18.1+ 5.8 (range 12–30 years)].

Over-production of PRL was controlled by pharmacological therapy with dopamine agonist (cabergoline) in 30 patients affected by PRLoma (50% of patients affected by PRLomas). One patient had to stop cabergoline therapy because she manifested hypotension and headache. Transphenoidal resection of pituitary adenomas was performed in 11 patients (14.67% of pituitary tumour-affected patients): 2 were ACTH-secreting tumours, 1 was a GH-secreting tumour, 2 were non-secreting adenomas and 6 were macro-PRLomas whose PRL over-secretion could not be controlled by pharmacological therapy.

Principal characteristics and treatments of pituitary tumours in our series are reported in Table 5.

**Table 5:** Main characteristics and treatments of pituitary tumours in our series of MEN1 patients

Pituitary tumours	Patients n. (%)	Clinical presentation			Surgery		Pharmacological treatment Dopamine agonist (Cabergoline) n. (%)
		FTs n. (%)	NFAs n. (%)	Combination of FTs n. (%)	No surgery n. (%)	Transphenoidal resection n. (%)	
Total patients with pituitary tumours	75 (51.72)%)	62 (42.76%) - 59 PRLomas (40.69%) - 3 corticotropinoma (2.07%)	12 (8.28%)	1 (0.69%) - 1 PRLoma-1 somatotropinoma (0.69%)	64 (85.33%)	11 (14.67%)	30 (40%)
A pituitary tumour as first clinical manifestation	25 (17.24%)	25 (17.24%) - 24 PRLomas (16.55%) - 1 corticotropinoma (0,69%)	0	0	18 (24%)	7 (9.33%)	10 (13.33%)
Index cases (probands)	54 (37.24%)	44 (30.34%) - 41 PRLomas (28.28%) - 3 corticotropinoma (2.07%)	9 (6.21%)	1 (0.69%) - 1 PRLoma-1 somatotropinoma (0.69%)	44 (58.67%)	10 (13.33%)	17 (22.67%)
Relatives	21 (14.48%)	18 (12.41%) - 18 PRLomas (12.41%)	3 (2.07%	0	20 (26.67%)	1 (1.33%)	13 (17.33%)

Footnotes: *FTs* Functioning tumours, *NFAs* non-functioning adenomas, *PRLoma* Prolactinoma

Percentages of clinical presentation were calculated with respect to MEN1 clinically affected patients (145). Percentages of surgery and pharmacological treatment were calculated with respect to pituitary adenoma affected patients (75)

### Carcinoids

Seventeen patients (14 women and 3 men) presented bronchopulmonary carcinoids (11.72% of MEN1 affected patients) with a mean age of tumour diagnosis of 47.3+ 12.5 years (range 26–73 years). One man showed a well-differentiated lung carcinoid at the age of 49 years as the first manifestation of MEN1 (0.69% of MEN1 affected patients); he was clinically and genetically diagnosed with MEN1 at the age of 50 after showing a well-differenced gastric NF-NET and a micro-PRLoma (interestingly at the age of 59 he had not manifested PHPT yet).

No thymic carcinoids were described in our series. Twenty-nine patients underwent surgical ablation of thymus, at the same time of parathyroid surgery, to prevent the development of thymus carcinoids.

### Skin lesions

Forty-four patients (30 women and 14 men) presented MEN1-typical skin lesions (30.34% of affected patients). Distribution and combinations of skin lesions is reported in Table 1. Lipomas (single or multiple) were the most common skin lesion (37/53) affecting 37 patients (25.52% of MEN1 affected patients and 69.81% of all skin lesions), both alone (20 cases) or in combination with other skin lesions (7 cases; 5 with angiofibromas, one with angiomas and one with angiofibromas and fibromas).

Lipomas were the first clinical manifestation of MEN1 in 7 cases (4.83% of affected patients) with a mean age of onset of 21.9 + 11.5 years (range 9–39 years); 3 were index cases (mean age 33.7 + 6.2 years; range 25–39 years) and 4 were relatives (mean age 13.0 + 4.3 years; range 9–20 years). Mean age of MEN1 diagnosis of these 7 patients was 27.2 + 13.5 years (range 7–39 years); mean age of MEN1 diagnosis of index cases was 37.7 + 3.4 years (range 33–41 years) while in relatives it was 16.7 + 11.6 years (range 7–33 years).

### Adrenocortical tumours/lesions

Twenty-seven patients (22 women and 5 men) were affected by tumours/lesions of the adrenal glands (18.62% of affected patients), as detailed reported in Table 1.

Mean age of tumour/hyperplasia diagnosis was 47.0 + 12.4 years (range 31–62 years), while mean age at MEN1 diagnosis was 39.1 + 12.2 years (range 21–73 years).

Adrenocortical adenoma/hyperplasia never resulted to be the first MEN1 manifestation in our series of patients.

### *MEN1* mutational analysis

One hundred forty-nine patients (90.3%) resulted to bear a *MEN1* point or an intra-exon small frameshift mutation, within the coding region or the splicing sites of the gene, identified by PCR-based Sanger’s sequencing. Seventy-eight of them were index cases (58 familial cases from 47 pedigrees and 20 single cases), while 71 were first-degree relatives of a mutation carrier, from 36 pedigrees (20 were still asymptomatic at the time of this study and they were excluded from genotype-phenotype association analyses).

One family [5] members: 1 index case and four relatives (one still asymptomatic) resulted carriers of two different inactivating *MEN1* mutations, one in exon 4 (Leu249Pro missense mutation) and one in exon 8 (g.1181delC frameshift mutation), both located on the same *MEN1* allele and inherited from the father of the index case.

Four patients (from two pedigrees) were identified as carriers of a large intragenic deletion, spanning more than one entire exon, by MPLA. Two other patients (brother and sister) resulted carriers of a familial predisposing haplotype by microsatellite analysis at 11q13 locus [5].

Ten patients (three familial cases from the same pedigree and seven single cases) resulted negative to *MEN1* mutation sequencing analysis, and were not further analysed by other screening techniques; they were all clinically diagnosed with MEN1.

Genetic test allowed to identify a total of 34 mutations carriers who were still asymptomatic at the time of MEN1 genetic diagnosis. Twenty of them were still asymptomatic at the time of this study. Fourteen [genetically diagnosed at mean age of 20.1 + 9.6 years (range 7–33 years)] developed the first clinical manifestation (mean age 24.1 + 7.7 years; range 14–34 years), after genetic diagnosis and during the time of the study.

Table 6 resumes the distribution of mutation types in our patients. A total of 59 different mutations were described, including two different mutations carried by one family (double mutation) and two large intra-genic deletions spanning more than one exon. Frameshift mutations (22) were the most common (37.93%); one frameshift mutation was carried by two unrelated pedigrees. We identified 17 different missense mutations (29.31%); three of them were, respectively, carried by: 1) two families and one single case; 2) two families; 3) two families. Six different nonsense mutations were reported (10.34%); two of them were carried by 3 pedigrees and one single case, and by one family and 2 single cases, respectively. Total identified splicing site mutations were 10 (17.24%); one of them affecting two different unrelated pedigrees.

**Table 6:** Distribution of *MEN1* mutation types in our MEN1 patients

Mutation type	Number of different mutations (number of mutations carried by more than one family/case)	Number of pedigrees (total cases)	Number of single cases	Total number of patients
Frameshift mutations	22 (1)	17 (46)	6	52
Missense mutations	17 (3)	15 (36)	6	42
Nonsense mutations	6 (2)	7 (18)	4	22
Splicing site mutations	10 (1)	7 (24)	4	28
Double mutation (frameshift and missense)	1	1 (5)	0	5
Large intra-genic deletions	2	2 (4)	0	4
Predisposing familial 11q13 haplotype	n.a.	1 (2)	0	2
Patients without an identified MEN1 mutation (negative Sanger’s sequencing analysis)	n.a.	1 (3)	7	10
Total	58	52 (138)	27	165

Footnote**:** *N.A*. Non-applicable

Distribution of mutations along the *MEN1* exons and exon-intron junctions is reported in Table 7.

**Table 7:** Distribution of mutations along coding region and splicing sites of the *MEN1* gene

Exon/intron	Number of different mutations (number of mutations carried by more than one family/case)	Number of pedigrees (total cases)	Number of single cases	Total number of patients	Type of mutation (number of different mutations; total number of patients)
Exon 2	9 (1)	7 (19)	3	22	Frameshift (5; 15) Missense (3; 5) Splicing site (1; 2)
Intron 2	1	0	1	1	Splicing site (1; 1)
Exon 3	6 (0)	4 (10)	2	12	Frameshift (2; 2) Missense (4; 10)
Intron 3	1	0	1	1	Splicing site (1; 1)
Exon 4	2 (1)	3 (4)	0	4	Missense (2; 4)
Intron 4	2 (1)	4 (18)	0	18	Splicing site (2; 18)
Exon 5	1	1 (3)	0	3	Frameshift (1; 3)
Intron 5	3 (0)	1 (2)	2	4	Splicing site (3; 4)
Exon 6	2 (0)	2 (5)	0	5	Frameshift (1; 2) Missense (1; 3)
Intron 6	0	0	0	0	n.a.
Exon 7	1	0	1	1	Frameshift (1; 1)
Intron 7	1	1 (2)	0	2	Splicing site (1; 2)
Exon 8	3 (0)	2 (8)	1	9	Frameshift (2; 5) Missense (1; 4)
Intron 8	0	0	0	0	n.a.
Exon 9	10 (3)	11 (30)	5	35	Frameshift (3; 8) Nonsense (2; 12) Missense (5; 15)
Intron 9	0	0	0	0	n.a.
Exon 10	12 (1)	10 (23)	4	27	Frameshift (7; 16) Nonsense (4;10) Missense (1; 1)
Double mutation	2 (0)	1 (5)	0	5	Frameshift exon 8 Missense exon 4
Total	56 (7)	47 (129)	20	149	Frameshift (23; 57*^§^) Nonsense (6; 22^#^) Missense (18; 47*^¥^) Splicing site (9; 28^ɸ^)

Footnote**:** Only point and intra-exon small frameshift mutations were included in this table. Intron mutations are all splicing site mutations and they are all located near the intron-exon junction affecting splicing regulatory sequences. *N.A*. non-applicable. * Both these numbers include five individuals bearing two different mutations on the same *MEN1* allele. ^§^This number includes five still asymptomatic carriers. ^#^This number includes four still asymptomatic carriers. ^¥^This number includes five still asymptomatic carriers. ^ɸ^ This number includes four still asymptomatic carriers

Exons 2, 9 and 10 resulted to be the three most mutated exons, with 9, 10 and 12 different mutations, respectively. Exons 9 and 10, which encodes nuclear localization signals (NLSs) of menin, are prevalently affected by frameshift and nonsense mutations which create a premature STOP codon and a truncated menin protein missing one or more NLSs and unable to react the nucleus. Missense mutations in exon 9 (codons 396–450) are all concentrated between codons 414–444, affecting binding sites of JUND (codons 323–428), NM23H1 (codons 1–486), RPA2 (codons 286–448), HDAC1 (codons 145–450) and CHES1 (codons 428–610). Splicing sites mutations are prevalently located in introns 4 and 5.

We analysed also the distribution of tumours/lesions with respect to different mutations, and any possible correlation between genotype and clinical phenotype (including in the analyses 129 symptomatic patients bearing a point or frameshift mutation, 4 patients carrying a large intra-genic deletion and 2 patients presenting a familial 11q13 predisposing haplotype). Table 8 reports tumours distributions with respect to *MEN1* mutation type, while Table 9 with respect to mutated exon/intron.

**Table 8:** Distribution of MEN1-related tumours/lesions with respect to *MEN1* type of mutations

Tumour/lesion (n.)	FS n. (%)	MS n. (%)	NS n. (%)	SP n. (%)	FS + MS n. (%)	Intragenic large deletions	11q13 familial haplotype	No identified *MEN1* mutation*
PHPT (139)	47 (100%)	37 (100%)	17 (94.44%)	22 (91.67%)	3 (75%)	4 (100%)	2 (100%)	7 (70%)
Symptomatic (54)	12 (25.53%)	18 (48.65%)	8 (44.44%)	9 (37.50%)	1 (25%)	3 (75%)	1 (50%)	2 (20%)
Asymptomatic (85)	35 (74.47%)	19 (51.35%)	9 (50%)	13 (54.17%)	2 (50%)	1 (25%)	1 (50%)	5 (50%)
GEP-NETs (86)	32 (68.09%)	16 (43.24%)	12 (66.67%)	13 (54.17%)	2 (50%)	4 (100%)	2 (100%)	5 (50%)
pNFTs (28)	12 (25.53%)	6 (16.22%)	3 (16.67%)	4 (16.67%)	1 (25%)	1 (25%)	0	1 (10%)
PPoma (1)	0	1 (2.70%)	0	0	0	0	0	0
pNFT-gastric NF NET (1)	0	0	0	1 (4.17%)	0	0	0	0
Gastrinomas (28)	7 (14.89%)	3 (8.11%)	5 (27.78%)	7 (29.17%)	1 (25%)	2 (50%)	0	3 (30%)
Insulinomas (13)	6 (12.77%)	2 (5.41%)	3 (16.67%)	1 (4.17%)	0	0	0	1 (10%)
VIPomas (2)	1 (2.13%)	1 (2.70%)	0	0	0	0	0	0
Gastrinoma-insulinoma (3)	2 (4.26%)	1 (2.70%)	0	0	0	0	0	0
Gastrinoma-glucagonoma (1)	0	1 (2.70%)	0	0	0	0	0	0
pNFTs-gastrinoma (9)	4 (8.51%)	1 (2.70%)	1 (5.56%)	0	0	1 (25%)	2 (100%)	0
Pituitary adenomas (75)	24 (51.06%)	19 (51.35%)	10 (55.56%)	11 (45.83%)	3 (75%)	1 (25%)	1 (50%)	6 (60%)
NFAs (12)	4 (8.51%)	2 (5.41%)	3 (16.67%)	2 (8.33%)	0	0	0	1 (10%)
PRLomas (59)	18 (38.30%)	17 (45.95%)	6 (33.33%)	9 (24.32%)	3 (75%)	1 (25%)	1 (50%)	4 (40%)
Corticotropinomas (3)	1 (2.13%)	0	1 (5.56%)	0	0	0	0	1 (10%)
PRLoma-somatotropinoma (1)	1 (2.13%)	0	0	0	0	0	0	0
Bronchopulmonary carcinoids (17)	5 (10.64%)	3 (8.11%)	2 (11.11%)	6 (25%)	0	0	0	1 (10%)
Skin lesions								
Lipomas (37)	16 (34.04%)	5 (13.51%)	2 (11.11%)	7 (29.17%)	3 (75%)	3 (75%)	0	1 (10%)
Angiomas/angiofibromas/fibromas (13)	3 (6.38%)	5 (13.51%)	2 (11.11%)	1 (4.17%)	1 (25%)	1 (25%)	0	0
Adrenal gland tumours/lesions (27)	9 (19.15%)	8 (21.62%)	3 (16.67%)	3 (12.5%)	2 (50%)	1 (25%)	0	1 (10%)

Footnotes: *N*. number, *FS* Frameshift, *MS* Missense, *NS* nonsense, *SP* Splicing site, *PHPT* Primary hyperparathyroidism, *GEP-NETs* Gastro-entero-pancreatic neuroendocrine tumours, *pNFTs* Pancreatic non-functioning tumours, *NF NET* Non-functioning neuroendocrine tumours, *VIP* Vasoactive intestinal peptide, *NFAs* Non-functioning adenomas

Percentages were calculated with respect to the number of any mutation type in symptomatic patients (47 frameshifts, 18 nonsense, 37 missense, 24 splicing site, 4 double mutation, 4 large intragenic deletions and 2 11q13 predisposing familial haplotype)

*Patients analysed only by PCR-based Sanger’s sequencing

**Table 9:** Distribution of MEN1-related tumours/lesions with respect to *MEN1* mutated exon/intron

Tumour/lesion (n.)	Exon 2 n. (%)	Intron 2 n. (%)	Exon 3 n. (%)	Intron 3 n. (%)	Exon 4 n. (%)	Intron 4 n. (%)	Exon 5 n. (%)	Intron 5 n. (%)	Exon 6 n. (%)	Intron 6 n. (%)	Exon 7 n. (%)	Intron 7 n. (%)	Exon 8 n. (%)	Intron 8 n. (%)	Exon 9 n. (%)	Intron 9 n. (%)	Exon 10 n. (%)
PHPT	18 (100%)	1 (100%)	11 (100%)	1 (100%)	4 (100%)	15 (93.75%)	3 (100%)	3 (75%)	5 (100%)	n.a	1 (100%)	1 (50%)	6 (100%)	n.a	31 (100%)	n.a	23 (95.83%)
Symptomatic	7 (38.89%)	0	6 (54.55%)	1 (100%)	3 (75%)	4 (25%)	1 (33.33%)	3 (75%)	1 (20%)	n.a	0	1 (50%)	2 (33.33%)	n.a	10 (32.26%)	n.a	8 (33.33%)
Asymptomatic	11 (61.11%)	1 (100%)	5 (45.45%)	0	1 (25%)	11 (68.75%)	2 (66.67%)	0	4 (80%)	n.a	1 (100%)	0	4 (66.67%)	n.a	21 (67.74%)	n.a	15 (62.5%)
GEP-NETs	12 (66.67%)	1 (100%)	7 (63.64%)	1 (100%)	1 (25%)	9 (56.25%)	1 (33.33%)	0	2 (40%)	n.a	1 (100%)	1 (100%)	5 (83.33%)	n.a	18 (58.06%)	n.a	14 (58.33%)
pNFTs	6 (33.33%)	0	1 (9.09%)	0	1 (25%)	3 (18.75%)	0	0	0	n.a	0	0	2 (33.33%)	n.a	6 (19.35%)	n.a	6 (25%)
PPoma	0	0	1 (9.09%)	0	0	0	0	0	0	n.a	0	0	0	n.a	0	n.a	0
pNFT-gastric NF NET	0	0	0	0	0	1 (6.25%)	0	0	0	n.a	0	0	0	n.a	0	n.a	0
Gastrinomas	1 (5.56%)	1 (100%)	2 (18.18%)	0	0	5 (31.25%)	0	0	0	n.a	0	1 (100%)	2 (33.33%)	n.a	4 (12.90%)	n.a	6 (25%)
Insulinomas	2 (11.11%)	0	3 (27.27%)	1 (100%)	0	0	1 (33.33%)	0	0	n.a	0	0	0	n.a	4 (12.90%)	n.a	1 (4.17%)
VIPomas	1 (5.56%)	0	0	0	0	0	0	0	0	n.a	0	0	0	n.a	0	n.a	1 (4.17%)
Gastrinoma-insulinoma	1 (5.56%)	0	0	0	0	0	0	0	0	n.a	1 (100%)	0	0	n.a	1 (3.23%)	n.a	0
Gastrinoma-glucagonoma	0	0	0	0	0	0	0	0	1 (20%)	n.a	0	0	0	n.a	0	n.a	0
pNFTs-gastrinoma	1 (5.56%)	0	0	0	0	0	0	0	1 (20%)	n.a	0	0	1 (16.67%)	n.a	3 (9.68%)	n.a	0
Pituitary adenomas	8 (44.44%)	0	7 (63.64%)	1 (100%)	3 (75%)	5 (31.25%)	2 (66.67%)	3 (75%)	1 (20%)	n.a	1 (100%)	1 (50%)	2 (33.33%)	n.a	18 (58.06%)	n.a	12 (50%)
NFAs	1 (5.56%)	0	2 (18.18%)	0	0	1 (6.25%)	0	0	1 (20%)	n.a	0	0	0	n.a	3 (9.68%)	n.a	3 (12.5%)
PRLomas	7 (38.89%)	0	5 (45.45%)	1 (100%)	3 (75%)	4 (25%)	1 (33.33%)	3 (75%)	0	n.a	0	1 (50%)	2 (33.33%)	n.a	15 (48.39%)	n.a	8 (33.33%)
Corticotropinomas	0	0	0	0	0	0	0	0	0	n.a	1 (100%)	0	0	n.a	0	n.a	1 (4.17%)
PRLoma-somatotropinoma	0	0	0	0	0	0	1 (33.33%)	0	0	n.a	0	0	0	n.a	0	n.a	0
Bronchopulmonary carcinoids	2 (11.11%)	1 (100%)	1 (9.09%)	0	1 (25%)	4 (25%)	1 (33.33%)	0	0	n.a	1 (100%)	0	0	n.a	2 (6.45%)	n.a	3 (12.5%)
Skin lesions																	
Lipomas	5 (27.78%)	0	3 (27.27%)	0	0	6 (37.5%)	2 (66.67%)	1 (25%)	0	n.a	1 (100%)	0	2 (33.33%)	n.a	6 (19.35%)	n.a	4 (16.67%)
Angiomas/angiofibromas/fibromas	0	0	2 (18.18%)	0	0	0	0	1 (25%)	0	n.a	0	0	0	n.a	6 (19.35%)	n.a	3 (12.5%)
Adrenal gland tumours/lesions	4 (22.22%)	0	5 (45.45%)	1 (100%)	1 (25%)	0	0	2 (50%)	0	n.a.	0	0	1 (16.67%)	n.a.	6 (19.35%)	n.a.	3 (12.5%)

Footnotes: *N* number, *PHPT* Primary hyperparathyroidism, *GEP-NETs* Gastro-entero-pancreatic neuroendocrine tumours, *pNFTs* pancreatic non-functioning tumours, *NF NET* Non-functioning neuroendocrine tumours, *VIP* Vasoactive intestinal peptide, *NFAs* Non-functioning adenomas, *N.A* non-applicable (no patients bearing these mutations)

Percentages were calculated with respect to the number of symptomatic patients for any mutated exon or intron

Statistical analyses did not evidence any significant difference neither between disease age of onset and *MEN1* four main mutation types or mutation localization, nor in the distribution of PHPT and pituitary tumours between different *MEN1* mutation types and localization.

Statistical analyses showed a significantly higher percentage of GEP-NETs in patients bearing a frameshift mutation (68.09%) with respect to missense mutations (43.24%; χ^2^ = 5.22, *p* = 0.022); however, not significant association was found in patients bearing a nonsense mutation (66.67%) versus patients with a missense mutation (43.24%; χ^2^ = 2.66, *p* = 0.103), suggesting that the only one reported positive association could be only an accidental statistical association. Indeed, the specific analysis of intra-familial MEN1 clinical phenotypes, age of onset, and disease penetrance (in 36 pedigrees for which we collected more than one affected member) highlighted a high clinical variability and a personal disease presentation even in the presence of the same mutation, thus, excluding any possible direct genotype-phenotype correlation.

## Discussion

The institution (and continuous updating) of patients’ large specific registries or databases is very useful in the management of rare diseases, like MEN1. Indeed, the collection of clinical, biochemical and genetic characteristics of unselected patients represents a good approach to increase the knowledge on epidemiological aspects of the disease, and natural course and prognosis of single manifestations of the syndrome.

During the last decade, important national MEN1 databases favoured the study of epidemiological, diagnostic, clinical and therapeutic aspects of MEN1-associated tumours, such as the DutchMEN1 Study Group (DMSG), the Groupe d’Etude des Tumeurs Endocrines (GTE), the multicentre database of the MEN1 Consortium of Japan [6], and the Italian MEN1 database [7].

Here, we reported the results of the analysis of a large database of Florentine MEN1 patients from the Referral Centre for Hereditary Endocrine Tumours of the “Regione Toscana”, comparing our data with the previously published. The availability of the Referral Centre allowed the strict interaction of healthcare specialists in different areas of NETs and, thus, granted the collection of extensive data and a continuous long-term follow-up of patients, as well as the possibility to perform genetic test and to associate genetic and clinical data.

Given the autosomal dominant partner of inheritance, MEN1 syndrome manifests an equal gender distribution, however, in our database a greater predominance (64.2% vs. 35.8%) of female patients was detected, confirming what had been previously shown in similar studies carried out in France, the Netherlands, Japan and Italy [6–9].

The analysis of our MEN1 database confirmed PHPT as the most common manifestation of the syndrome, reaching a penetrance of over 95% after the age of 55, followed by GEP-NETs (about 60%) and pituitary tumours (about 52%), respectively. These data were in accordance with those previously reported by the Japan database [6], and found in the Italian MEN1 database [7], which includes also part of our Florentine patients.

Distribution of GEP-NETs in our database was in accordance with previously published data [1] for all tumour types; insulinoma resulted to be higher in Japanese patients (22%) with respect to Western countries (10%) [1] and our study (about 11%).

Regarding pituitary tumours, our patients showed a higher prevalence of PRLoma (over 41%) with respect to data reported in the MEN1 International guideline (20%) [1], in the DMSG database (16%) [8] and in the GTE cohort (30%) [10].

In our cohort PHPT was prevalently treated by TPT with parathyroid tissue autograft, conversely to a cohort study of the DMSG in which STP with bilateral trans-cervical thymectomy was the procedure of choice [11], and PHPT patients from the MEN1 Japan database which were operated, more than a half, by single gland parathyroidectomy [6]. TPT granted a lower rate of persistence and a longer PHPT-free post-operatory period, than SPT and PPT. A very low percentage of operated patients (5.1%) manifest post-surgical permanent hypoparathyroidism that has been pharmacologically mitigated over the years.

General mean age of diagnosis of the three main clinical MEN1 manifestation in our patients was about 15 years (reaching up to 20 years in case of gastrinomas) earlier than sporadic counterparts; due to natural characteristics of MEN1 but also favoured by the periodic tumour-surveillance screening program and constant follow-up of all MEN1 affected patients and mutation carriers. MEN1-associated tumours have an earlier age of onset, with respect to sporadic counterparts, and often they present a more aggressive course and behaviour; an early diagnosis, followed by an early surgical and/or pharmacological intervention, is the gold standard for reducing morbidity and mortality. In this light, the institution of patients’ database helps in better setting and programming a continuous monitoring and follow-up of patients and periodical screenings of both affected and asymptomatic mutation carriers, increasing the reduction of morbidity and mortality of MEN1. Morbidity in our patients was principally due to a hormone over-secretion (i.e gastrin leading to ZES and causing ulcers and bleeding; PRL responsible for amenorrhea, oligomenorrhea and/or galactorrhea in women and impotence, infertility, decreased libido in men; insulin provoking hypoglycemia, etc), while mortality was caused, prevalently, by malignant progression and metastases of not early detected gastrinomas and carcinoids. Data from the DMSG evidenced that half of patients, diagnosed with liver metastases from duodenopancreatic NETs, died precociously, in a median follow-up of 4 years since tumour discovery, prompting the importance of an early diagnosis and a constant periodic clinical surveillance [12]. Indeed, data from our database highlighted that all the precociously deceased patients were characterised by a late diagnosis of MEN1 (about 15 years after the occurrence of the first clinical manifestation), and they were already presenting, at the time of diagnosis, malignancies (with or without metastasis) and/or severe tumour-related consequences untreatable by current available surgical and/or medical options. Long-term unrecognised and untreated gastrinomas and MEN1-related malignancies were the most common causes of death among our patients, confirming data from a GTE study which indicated duodenopancreatic and thymic NETs as responsible for increased risk of death in MEN1 patients [13].

Data from our database confirmed that biochemical screenings for hormone over-production are able to anticipate the diagnosis of MEN1 of over 10 years with respect to imaging methods, for functioning tumours; radiological screenings are the only effective diagnostic method for NF-NETs. Given the high malignant potential of pancreatic NFTs and their frequency in MEN1 patients, routine radiological surveillance of entero-pancreatic tract is mandatory. Our screening protocol consists in performing abdominal RMI of CT scan every 3 years in mutation carriers and in MEN1 affected individuals by the age of 20, associated with an intermediate pancreatic eco-endoscopy at 18 months, or, alternatively, an abdominal RMI of CT scan every 2 years without the pancreatic eco-endoscopy. In our series of patients, these imaging approaches granted the early recognition of 39 NF pancreatic NETs and a gastric NF-NET, and the pre-operatory localization of tumours. None of our patients died for an undiscovered NF-GEP-NETs, confirming the importance of these applied diagnostic procedures, accordingly also with suggestion of the GTE group [14].

The genetic test is the only one that grants a real early diagnosis, allowing the identification of mutation carriers, within mutated pedigrees, at their still asymptomatic level, and decades before the appearance of any biochemical valuable alteration. The application of genetic test in our patients allowed, indeed, the identification of 34 asymptomatic mutation carriers, 14 of them manifested their first clinical manifestation of MEN1 a mean of about 4 years after the genetic diagnosis; the constant diagnostic screening after genetic test granted the early recognition of the clinical manifestations and favoured a very early therapeutic intervention. In the last two decades, thanks to early genetic diagnosis and identification of mutation carriers at a very young age it has been possible to analyse MEN1 features and manifestations also in young individuals. For years, evidence and data of MEN1 in childhood and adolescents had been derived mainly from case reports. Recently, thanks to the establishment of national databases and collection of large series of patients, two studies, one from the GTE database [15] and one from this Florentine database [16], have investigated clinical, therapeutic and genetic aspects of MEN1 in children and adolescents (respectively before the age of 21 and 20 years), favouring the collection of important data regarding the management of this syndrome in young individuals.

The progressive application of *MEN1* genetic test worldwide notably decreased MEN1-associated morbidity and mortality. Unfortunately, MEN1 syndrome shows no direct correlation between genotype and phenotype [17, 18]. Only one study [19] on the GTE cohort showed that survival rate of mutated MEN1 patients was significantly lower among carriers of a *MEN1* mutation affecting binding sites with JunD, presenting a 2-fold higher death risk of MEN1-associated tumours; no association was found between mutations and other peculiar phenotypic features. The analysis of our MEN1 patients’ database, as well as that of the Italian MEN1 database [18], confirmed the lack of a direct correlation between a specific mutation, mutation type or mutated gene region with clinical manifestations and MEN1 phenotypes, not allowing the setting of personalised screening and therapeutic programs. The detailed intra-familial analysis of clinical phenotype, age of tumour onset, multiple tumour association, disease penetrance, severity, course and prognosis in all our pedigrees with more than one affected member confirmed the total absence of correlation between these characteristics and the *MEN1* mutation. Only a single positive association between development of a GEP-NET and frameshift mutations was reported, with respect to missense mutations. However, this association was not replicated for another mutation type with similar gap in percentage of tumour occurrence; this suggested that the positive association could be only an accidental statistical association. This datum needs to be verified or denied by further studies in broad and different MEN1 series of patients. The GTE research group demonstrated, in MEN1 pedigrees from its database, an intra-familial heritability for pituitary (64%), adrenal (65%) and thymic NETs (97%), progressively decreasing along parental degree distance [20]. The progressive decrease along generations of intra-familial clinical hereditability and, mostly of all, the absence of direct genotype-phenotype correlation prompt the hypothesis of an important, direct role of other modifying factors in the determination of individual MEN1 tumorigenesis. Since differences in MEN1 phenotype have been reported also in identical twins, epigenetics factors, such as microRNAs and histone modifications, are, currently, considered as the most probable responsible determinants, presumably triggered by environmental factors, to define the MEN1 phenotype in patients bearing the same *MEN1* mutation. Identification and study of these factors, thanks to the availability of patients’ databases and tissue banks, are mandatory for a complete comprehension of MEN1 tumorigenesis, and for the development of new target diagnostic and therapeutic strategies.

## Conclusions

In conclusion, our long-term clinical practice (as Referral Centre for endocrine inherited tumour syndromes), together with the institution of an over 25 year-lasting MEN1 patients’ database, highlighted that the collection of data (including a detailed family and personal clinical and therapeutic history), the performance of the genetic test in patients and first-degree relatives, as well as the continuous follow up are essential for a correct and early diagnosis and for granting patients the best available diagnostic and therapeutic management.

## Abbreviations

ACTH: Corticotropin (adrenocorticotropic hormone)
GEP: Gastro-entero-pancreatic
GEP-NETs: Gastro-entero-pancreatic neuroendocrine tumours
GH: Somatitropin (growth hormone)
MEN1: Multiple endocrine neoplasia type 1
MPLA: Multiplex ligation-dependent probe amplification
NETs: Neuroendocrine tumours
NF-NETs: Non-functioning neuroendocrine tumours
NFTs: Non-functioning tumours
NLSs: Nuclear localization signals
PHPT: Primary hyperparathyroidism
PP: Pancreatic polypeptide
PPT: Partial parathyroidectomy
PRL: Prolactin
PTH: Parathyroid hormone
SD: Standard deviation
SPT: Subtotal parathyroidectomy
SSAs: Somatostatin analogues
TPT: Total parathyroidectomy
VIP: Vasoactive intestinal polypeptide
ZES: Zollister Ellison syndrome
